# TRPA1 Contributes to FGFR2c Signaling and to Its Oncogenic Outcomes in Pancreatic Ductal Adenocarcinoma-Derived Cell Lines

**DOI:** 10.3390/cancers16030609

**Published:** 2024-01-31

**Authors:** Vanessa Mancini, Salvatore Raffa, Alessandra Fiorio Pla, Deborah French, Maria Rosaria Torrisi, Danilo Ranieri, Francesca Belleudi

**Affiliations:** 1 Department of Clinical and Molecular Medicine, Sapienza University of Rome, 00161 Rome, Italy; vanessa.mancini@uniroma1.it (V.M.); salvatore.raffa@uniroma1.it (S.R.); deborah.french@uniroma1.it (D.F.); mara.torrisi@uniroma1.it (M.R.T.); francesca.belleudi@uniroma1.it (F.B.); 2 Turin Cell Physiology Laboratory, Department of Life Sciences and Systems Biology, University of Turin, 10125 Torino, Italy; alessandra.fiorio@unito.it; 3 Dipartimento di Scienze della Vita, della Salute e delle Professioni Sanitarie, Università degli Studi “Link Campus University”, 00165 Rome, Italy

**Keywords:** FGFR2, TRPA1, PDAC, PKCε, EMT

## Abstract

**Simple Summary:**

Given the previously proposed oncogenic function of the high expression of the mesenchymal FGFR2c variant in PDAC-derived cells, in this work, we investigated the contribution of the TRPA1 channel in the FGFR2c/PKCε axis. Our results highlighted a pore-independent role of this channel in the FGFR2c-mediated enhancement of EMT and the invasive behavior of PANC-1 PDAC cells, proposing TRPA1 as a putative candidate for future target therapies in PDAC.

**Abstract:**

Fibroblast growth factor receptor (FGFR) signaling is a key modulator of cellular processes dysregulated in cancer. We recently found that the high expression of the mesenchymal FGFR2c variant in human pancreatic ductal adenocarcinoma (PDAC)-derived cells triggers the PKCε-mediated improvement of EMT and of MCL-1/SRC-dependent cell invasion. Since other membrane proteins can affect the receptor tyrosine kinase signaling, including transient receptor potential channels (TRPs), in this work, we investigated the role of TRPs in the FGFR2c/PKCε oncogenic axis. Our results highlighted that either the FGFR2c/PKCε axis shut-off obtained by shRNA or its sustained activation via ligand stimulation induces TRPA1 downregulation, suggesting a channel/receptor dependence. Indeed, biochemical molecular and immunofluorescence approaches demonstrated that the transient depletion of TRPA1 by siRNA was sufficient to attenuate FGFR2c downstream signaling pathways, as well as the consequent enhancement of EMT. Moreover, the biochemical check of MCL1/SRC signaling and the in vitro assay of cellular motility suggested that TRPA1 also contributes to the FGFR2c-induced enhancement of PDAC cell invasiveness. Finally, the use of a selective channel antagonist indicated that the contribution of TRPA1 to the FGFR2c oncogenic potential is independent of its pore function. Thus, TRPA1 could represent a putative candidate for future target therapies in PDAC.

## 1. Introduction

Pancreatic ductal adenocarcinoma (PDAC) is certainly among the most lethal tumors presenting gain-of-function mutations of KRAS, coupled to a wide spectrum of additional mutations targeting several intracellular signaling substrates and determining a plethora of clinical cancer subtypes [1,2,3]. Since therapies currently applied in PDAC, combining target and immunotherapy approaches with standard chemotherapy, are scarcely satisfactory [1,2,3], identifying new key signaling molecules for targeting has become a very urgent need in order to counteract PDAC development and progression.

Pancreatic cancer is also characterized by a pro-tumorigenic and desmoplastic stroma, in which a close crosstalk between cancerous and stroma cells is established, favoring tumor progression and chemoresistance [1,2,3,4,5]. The FGF/FGFR axis has been described as one of the critical signaling pathways involved in this crosstalk [6,7,8], pointing to it as a suitable therapeutic target for new therapies. 

Indeed, pancreatic tumor cells and tissues display different expression profiles of FGFRs (FGFR1-4) [7], and opposite expression trends of FGFR1 and FGFR2 have been recently described in PDAC-derived cell lines [9,10], which lead to the enhancement of alternative signaling pathways and the consequent acquisition of distinct tumor hallmarks [9,10]. Therefore, assessing the FGFR1/2 expression profile in each pancreatic tumor could significantly contribute to predicting tumor cell response to paracrine factors and therapies. The crucial role of FGFR2, in particular of its mesenchymal isoform FGFR2c in PDAC tumorigenesis, has been further strengthened by our recent evidence showing that, when highly expressed, this receptor drives a PKCε-mediated axis that enhances several oncogenic features, including EMT, dysregulated autophagy, and MCL-1/SRC-mediated cell invasion [10,11]. 

To make this scenario even more complex, recent evidence has led to the supposition that, in establishing cancer hallmarks, RTKs, including FGFRs, can enter into interplay with the Ca^2+^-specific transient receptor potential channels (TRPs) [12], which in turn appear to provide their contribution via either channel-dependent or channel-independent functions [13,14]. Indeed, the role of TRPs in cancer is still widely debated, and the molecular mechanisms of this involvement still remain largely unknown. 

At least in the case of lung cancer, a direct TRPA1–FGFR2 interaction has been demonstrated, which results in the inhibition of TRPA1 activity, due to a conformational change in TRPA-1, which in turn induces the ligand-independent activation of FGFR2 and the consequent sustained proliferation and cell invasion [15,16]. Given the importance of TRPs–FGFR2 interaction in this context, its relevance in other carcinomas cannot be excluded. In light of this, the challenge of this work was to assess whether the mainly expressed TRP in PDAC could participate in the establishment of the FGFR2c/PKCε-mediated axis and on its oncogenic outcomes. 

## 2. Materials and Methods

### 2.1. Cells and Treatments

The PANC-1 and MIA PaCa-2 cell lines (ATCC, Manassas, VA, USA) were cultured and silenced for FGFR2 and PKCε as reported [10]. 

For RNA interference and the consequent specific TRPA1 silencing, cells were transfected with a TRPA1 small interfering RNA (TRPA1 siRNA) sequence (Santa Cruz Biotechnology, Dallas, TX, USA; sc-44780) as reported [10]. For the inhibition of TRPA1 activity, cells were treated with a specific blocker of TRPA1 channels, A-967079 (MedChemExpress, Monmouth Junction, NJ, USA; HY-108463), as previously described [17]. For growth factor stimulation, cells were as previously described [10]. For the inhibition of proteasomal or lysosomal degradation activity, cells were incubated with 10 nM bortezomib (BZ) (Sigma-Aldrich, Saint Louis, MO, USA, Cat. N 5.04314) for the last 3 h or with 10 μM chloroquine (Sigma Aldrich, Cat. N. C6628) for the last 12 h of the 24 h FGF2 treatment.

### 2.2. Immunofluorescence

Cells were grown on coverslips and processed as previously reported [10]. Cells were then incubated with the following primary antibodies: monoclonal antibody anti-vimentin (Dako, Glostrup, Denmark; M0725) for 1 h at 25 °C. 

The primary antibodies were visualized, and images were taken, as previously described [10]. The quantitative evaluation of cell morphological changes in PANC-1 and MIA PaCa-2 cells was assessed by Fiji ImageJ software (version 1.54h) [18]. Cell circularity index was calculated as 4π × (area)/(perimeter)^2^, where a score of 1 corresponded to a circular shape, and smaller scores corresponded to a progressively elongated shape. Binary images were created by thresholding the images to remove background noise. Then, measurements were performed on the binary images considering, for each sample, 100 randomly chosen cells from 3 independent experiments [19]. Results are expressed as mean values ± SD.

### 2.3. Western Blot Analysis

Total lysates were resolved and blotted as previously reported [11]. The membranes were blocked with EveryBlot Blocking Buffer (Bio-Rad Laboratories, Hercules, CA, USA, 12010020) and incubated with anti-E-cadherin (GT311 GeneTex, Irvine, CA, USA), anti-vimentin (M0725, Dako, Glostrup, Denmark), anti-alpha-smooth muscle actin (1A4,Thermo Scientific, Rockford, IL, USA), anti-phospho-fibroblast growth factor receptor substrate 2 α (FRS2-α) (Tyr196) (Cell Signaling Technology, Beverly, MA, USA, #3864), the anti-phospho-sarcoma kinase (Src) family (Tyr416, D49G4) (Cell Signaling Technology, Beverly, MA; USA, #6943), anti-TRPC1 (Santa Cruz; #SC-133076) monoclonal antibodies or anti-p-MTOR (Ser 2448; Cell Signaling; 5536S), anti-p-p44/42 mitogen-activated protein kinase (MAPK) (p-ERK1/2) (Thr202/Tyr204; Cell Signaling; 9101S), anti-Bek (Santa Cruz Biotechnology; C17, sc-122), anti-p-S6K (ser 371, Cell Signaling, #9208), anti p-PKCε (Ser729, Abcam, Cambridge, UK; ab63387), anti-myeloid cell leukemia 1 (Mcl-1) (D35A5) (Cell Signaling; #5453), anti-TRPA1 (Abcam; #ab62053), and anti-TRPM8 (Abcam; #ab3243) polyclonal antibodies. The membranes were stripped as reported [11] and probed again with anti-p44/42 MAPK (ERK1/2) (Cell Signaling; 4695S), anti-S6K (Cell Signaling; #9202), anti-PKCε (Abcam; #ab124806), anti-FRS2 (H-91) (Santa Cruz Biotechnology; sc-8318), anti-α/β-tubulin (Cell Signaling; 2148S), anti-HSP90 (Proteintech Inc., Rosemont, IL, USA, 13171-1-AP), anti-MTOR (Cell Signaling; #2983S) polyclonal antibodies or anti-Src (Cell Signaling; #36D10), and anti-ACTB (Sigma-Aldrich; A5441) monoclonal antibodies for protein equal loading. Densitometric quantitative analysis was performed as previously described [11]. Mean values (±SD) were obtained from three different experiments, then normalized and expressed as the fold increase, with respect to the control value, and reported in graphs. All uncropped blots for each Western blot experiment are reported as Supplementary Materials.

### 2.4. Invasion Assay

Migration assay and quantitative analysis were assessed as reported [10]. 

### 2.5. Primers

Oligonucleotide primers were drowned with Primer-BLAST [20]. The following primers were used for the TRPA1 target gene: 5′-TAATGGGAAAGCCACCCCTC-3′ (sense) and 5′-GCACCTTCCCTTCTCCACTG-3′ (sense). For the 18S rRNA housekeeping, FGFR2b/KGFR and FGFR2c sequences were previously reported [10].

### 2.6. RNA Extraction and cDNA Synthesis

Total RNA was obtained using the TRIzol (Invitrogen, Waltham, MA, USA) and prepared as reported [10]. The total RNA concentration was evaluated by spectrophotometry; the c-DNA was obtained with the iScriptTM cDNA synthesis kit (Bio-Rad, 170-8891) according to the manufacturer’s protocol.

### 2.7. PCR Amplification and Real-Time Quantitation

Real-time PCR and gene expression quantitation were performed as previously described [21]. FGFR2c and FGFR2b target gene values were normalized to the value of the HFs and primary human keratinocyte cell line HaCaT, respectively. The mRNA levels were expressed as previously described [10]. 

### 2.8. Statistics

All quantitative data were analyzed using analysis of variance (ANOVA) to test for differences amongst all means. A Tukey’s multiple comparisons test was used to determine differences between selected groups. The *t*-test was applied in single comparisons. The significance levels were defined as *p*-values ≤ 0.05. All original western blots are presented in Supplementary File.

## 3. Results

### 3.1. TRPA1 Is a Possible Candidate for FGFR2c Interplay

**Figure 1:**
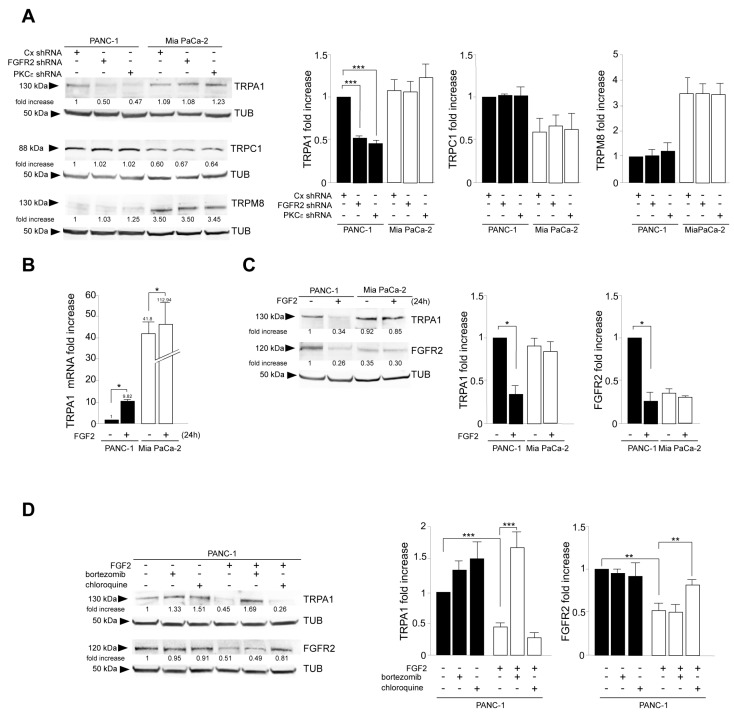
FGFR2c expression rate and its FGF2-mediated signaling impacts on TRPA1 expression. PANC-1 and MIA PaCa-2 cells were stably transfected with Bek/FGFR2 short hairpin RNA (shRNA), with PKCε shRNA, to obtain stable protein depletion. Stable transfection with control (Cx) shRNA was used as the negative control. (**A**) Western blot analysis showed that either FGFR2c or PKCε gene silencing modulated TRPA1 expression but not that of TRPC1 or TRPM8; the effect was evident exclusively in PANC-1 cells highly expressing FGFR2c. The densitometric analysis and the statistical evaluation were performed as reported in the Materials and Methods section. Results are expressed as mean value ± SD; *** *p* < 0.001. (**B**,**C**) PANC-1 and MIA PaCa-2 cells were stimulated with FGF2 for 24 h. (**B**) Real-time RT-PCR showed that ligand stimulation increased TRPA1 mRNA levels, especially in PANC-1 cells. Results are reported as mean ± SD from three different experiments in triplicate. Statistical analysis was performed, as reported in the Materials and Methods section; * *p* < 0.05. (**C**) Western blot analysis showed that only in PANC-1 cells, both TRPA1 and FGFR2c protein amounts were decreased in response to FGF2 stimulation. The densitometric analysis and the statistical evaluation were performed as reported above; * *p* < 0.05. (**D**) PANC-1 cells were stimulated with FGF2 for 24 h in the presence or not of the proteosome inhibitor bortezomib or in the presence of the lysosome inhibitor chloroquine. Western blot analysis showed that the FGF2-induced downregulation of TRPA1 was recovered in the presence of the proteosome inhibitor (bortezomib), while that of FGFR2c is recovered in the presence of the lysosome inhibitor (chloroquine) was not. Both inhibitors did not significantly impact TRPA1 or FGFR2c protein amounts in unstimulated cells. The densitometric analysis and the statistical evaluation were performed as reported above; ** *p* < 0.01; *** *p* < 0.001.

As a first step, we tried to highlight whether a possible TRP/FGFR2c dependence could exist in PDAC-derived cells. For this aim, we took advantage of cellular models of primary PDAC-derived PANC-1 and MIA PaCa-2 cell lines, previously chosen by us because of their opposite trends in the expression of the mesenchymal FGFR2c variant and very low amounts of the epithelial counterpart FGFR2b [10,22,23]. TRPA1, TRPC1, and TRPM8 were chosen for the investigation, as they are the TRPs mainly expressed in PDAC tumors and cells [17,24,25,26]. FGFR2c signaling repression was obtained by FGFR2 depletion via the stable transfection of FGFR2-specific short harpin RNA. The gene silencing approach was alternatively performed to repress the main FGFR2c downstream hub signaling molecule PKCε. The preliminary molecular analysis by real-time RT-PCR performed in untransfected cells confirmed their divergent expression of the mesenchymal FGFR2c isoform, as well as their negligible expression of the epithelial FGFR2b variant (Supplementary Figure S1A). The efficiency of a specific shRNA-induced gene silencing was then assessed by Western blot (Supplementary Figure S1B). Biochemical analysis performed in shRNA clones of both PANC-1 and MIA PaCa-2 cells showed that TRPA1 expression, but not that of TRPC1 or TRPM8, was decreased by either FGFR2 or PKCε depletion (Figure 1A). This effect was observed exclusively in PANC-1 cells (Figure 1A), further strengthening the possibility of its dependence on FGFR2c high expression. These results encouraged us to investigate the possibility of a channel/receptor link, at least in the case of TRPA1. For this aim, we assayed the effects of FGFR2c-sustained signaling on TRPA1 expression. PDAC cells were stimulated for 24 h with FGF2, the ligand which binds FGFR2c but not its epithelial counterpart, FGFR2b. Real-time RT-PCR analysis highlighted an increase in TRPA1 mRNA levels, especially in PANC-1 cells (about 10-fold increase) (Figure 1B), while the parallel biochemical analysis surprisingly showed an opposite repressive effect on the protein amount (Figure 1C). The decrease in the TRPA1 protein was detectable only in PANC-1 cells and was accompanied by a significant downregulation of FGFR2c, suggesting a possible receptor/channel common fate. By common fate, we mean that FGF2 stimulation could induce FGFR2c sorting to an endocytic degradative pathway, and TRPA1 could possibly follow it. This pathway could target the receptor and channel to the lysosomes, where both are degraded.

To check this possibility, Western blot analysis was performed after sustained stimulation with FGF2 in the presence of selective inhibitors for each intracellular degradative pathway, including the lysosomal and the proteosomal routes. The results revealed that the downregulation of TRPA1 can be ascribed to proteasome sorting, while that of FGFR2c is dependent on its targeting to lysosomes (Figure 1D). Overall, our results, even if they did not give indications about a possible FGFR2c/TRPA1 interaction, suggested that the high expression and the activation of FGFR2c are required for TRPA1 mRNA induction and its protein stability. Therefore, when the receptor undergoes massive downregulation by either sustained ligand stimulation or gene silencing, TRPA1 is possibly sorted to proteosomes and degraded.

### 3.2. TRPA1 Contributes to FGFR2c-Established Aberrant Signaling and to the Consequent Enhancement of EMT and Invasive Traits

We recently proposed that when highly expressed in PDAC cells, FGFR2c triggers an aberrant signaling transduced by PKCε, which contributes to simultaneously counteracting MTOR-dependent autophagy and enhancing EMT, directly converging and enhancing ERK1/2 signaling [10,11]. 

**Figure 2:**
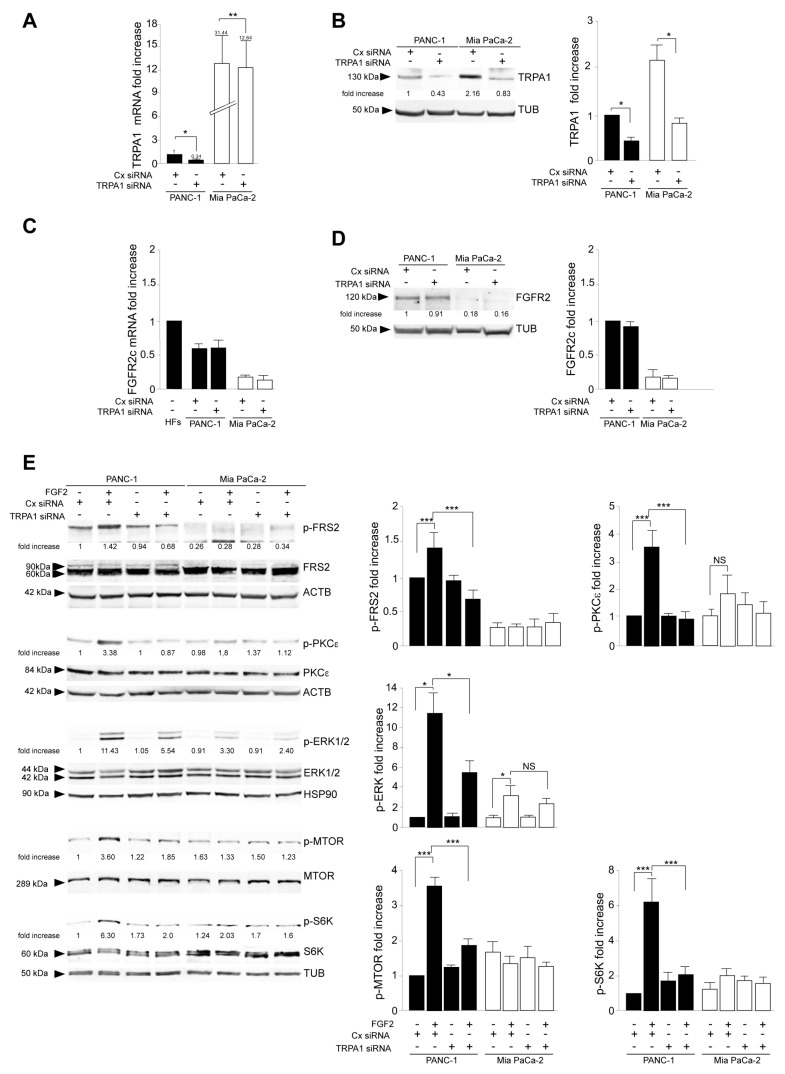
TRPA1 depletion interfered with FGFR2c oncogenic signaling. PANC-1 and MIA PaCa-2 cells were transiently transfected with TRPA1-specific siRNA or unrelated siRNA (Cx siRNA) as the negative control and then left untreated or stimulated with FGF2 to induce FGFR2c activation and signaling. The efficiency of gene silencing was assessed by real-time RT-PCR (**A**) and Western blot (**B**); * *p* < 0.05; ** *p* < 0.01. Biochemical analysis showed that TRPA1 depletion did not affect FGFR2c expression at either the mRNA (**C**) or protein (**D**) level. (**E**) Western blot analysis showed that only in PANC-1 cells, TRPA1 silencing repressed the FGF2-mediated phosphorylation of the FGFR2c signaling platform FRS2 and that of PKCε and ERK1/2, as well as that of MTOR and its substrate S6K. Results are expressed as mean value ± SD. The densitometric analysis and the statistical evaluation were performed as reported in the Materials and Methods section; * *p* < 0.05; *** *p* < 0.001.

To assess the contribution of TRPA1 on this aberrant signaling, TRPA1 gene silencing was performed in PANC-1 and MIA PaCa-2 cells using transient transfection with specific small interfering RNAs (siRNA). The transient transfection with unrelated siRNA (CxRNA) was performed as the negative control. The efficiency of gene silencing was assessed by real-time RT-PCR (Figure 2A) and Western blot (Figure 2B). Further biochemical analysis revealed that in the opposite way to what was observed in TRPA1 expression as a consequence of FGFR2c silencing, the transient depletion of the channel did not impact FGFR2c expression, either at the mRNA or the protein levels (Figure 2C,D). 

**Figure 3:**
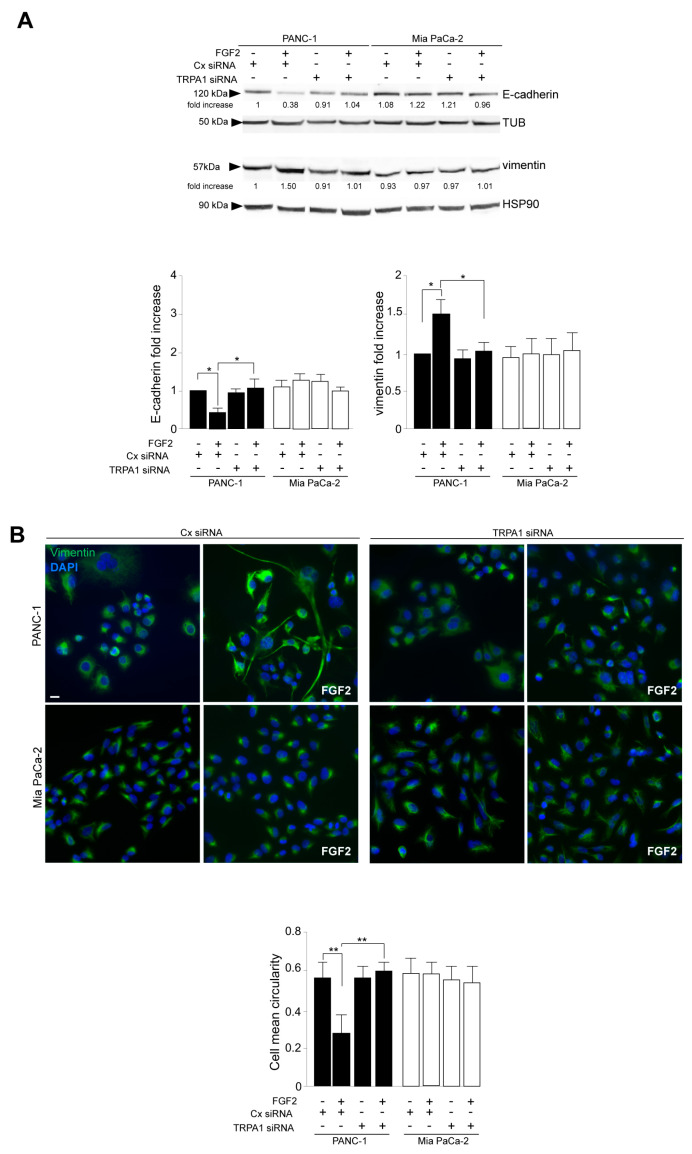
TRPA1 depletion impacts on the FGFR2c-mediated enhancement of the EMT phenotype in response to FGF2. PANC-1 and MIA PaCa-2 cells were transfected with TRPA1 siRNA or control (Cx) siRNA and left untreated or stimulated with FGF2 for 24 h, as shown above. (**A**) Western blot analysis showed that only in PANC-1 cells, the decrease in the epithelial marker E-cadherin and the increase in the mesenchymal marker vimentin induced by FGF2 stimulation are counteracted by TRPA1 gene silencing. E-cadherin and vimentin expressions did not significantly change in MIA PaCa-2 cells. Results are expressed as mean value ± SD. The densitometric analysis and the statistical evaluation were performed as reported in the Materials and Methods section; * *p* < 0.05. (**B**) Immunofluorescence analysis showed that the effects of FGF2 stimulation in terms of changes in cell morphology (detachment from each other and acquisition of a spindle shape) and increases in intensity of vimentin immunostaining, visible only in PANC-1 cells, appeared reversed by TRPA1 depletion. Bar: 10 μm. Quantitative analysis of cell circularity and the statistical evaluation were performed as reported in the Materials and Methods section; ** *p* < 0.01.

Western blot analysis, performed in cells stimulated with FGFR2c for early signaling activation, highlighted that TRPA1 silencing repressed the FGF2-mediated phosphorylation of the FGFR2c platform FRS2 and of PKCε and ERK1/2, as well as that of MTOR and its substrate S6K (Figure 2E), indicating a general negative impact on all FGFR2c-mediated oncogenic signaling pathways. In the second step, we focused our attention on the effects of TRPA1 depletion on the EMT phenotype that we previously showed was enhanced by the establishment of the FGFR2c/PKCε axis in cells highly expressing FGFR2c [10]. The biochemical analysis by Western blot revealed that the decrease in the epithelial marker E-cadherin and the increase in the mesenchymal marker vimentin, induced by FGF2 stimulation only in PANC-1 cells, appeared significantly counteracted by TRPA1 depletion (Figure 3A). A significant increase was also observed for α-SMA (Supplementary Figure S2A), which is a key EMT marker whose modulation was previously checked in PANC-1 cells [27]. TRPA1 depletion also counteracted the induction of Snail 1 (Supplementary Figure S2B), the main the transcription factor for pathological EMT [28,29], which we recently observed to be upregulated in PANC-1 cells as a consequence of the FGF2-mediated activation of FGFR2c [10]. A comparable effect was observed on changes in cell morphology (detachment from each other and acquisition of a spindle shape) and on the intensity of vimentin immunofluorescence staining, displayed by PANC-1 cells in response to FGF2 (Figure 3B). 

**Figure 4:**
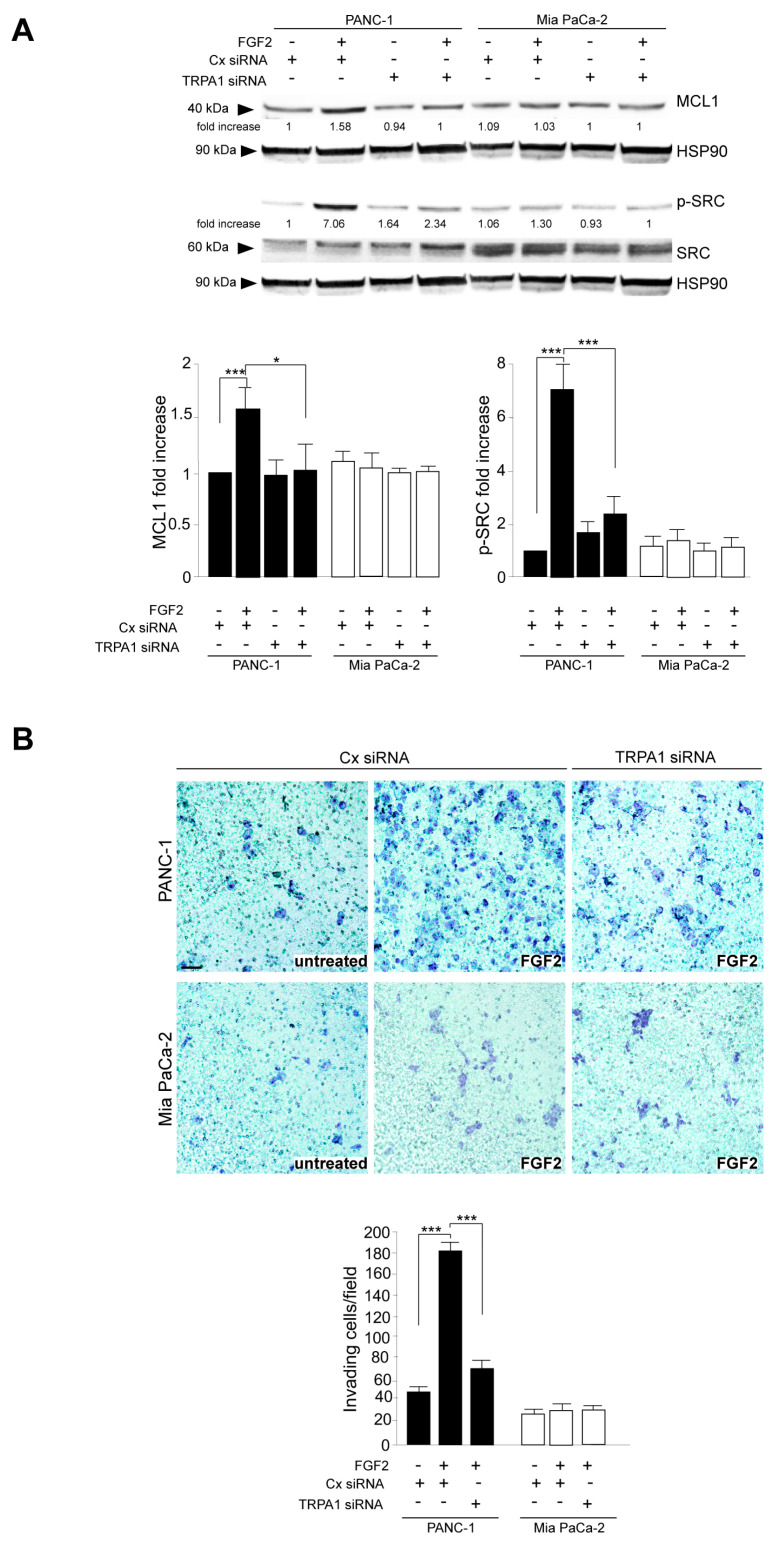
TRPA1 repression counteracts FGF2-mediated cell invasion. (**A**) PANC-1 and MIA PaCa-2 cells were transfected with TRPA1 or control (Cx) siRNA and left untreated or stimulated with FGF2 for 24 h, as shown above. Western blot analysis showed that the increases in MCL-1 levels and SRC phosphorylation, observed only in PANC-1 cells after FGF2 stimulation, were inhibited by TRPA1 gene silencing. The densitometric analysis and the statistical evaluation were performed as reported in the Materials and Methods section; * *p* < 0.05; *** *p* < 0.001. (**B**) PANC-1 and MIA PaCa-2 cells transfected with TRPA1 siRNA or with Cx siRNA, as described above, were seeded on Matrigel pre-coated Transwell Boyden chamber filters. After 24 h, FGF2 was added in the bottom chamber for 48 h to stimulate cell chemotaxis. Cell invasion in response to FGF2, visible only in PANC-1 cells, was strongly dampened by TRPA1 depletion. The quantitative analysis and the statistical evaluation were performed as reported in the Materials and Methods section; *** *p* < 0.001; bar: 50 μm.

The additional biochemical investigation revealed that the depletion of the TRPA1 protein also negatively impacted the increase in either the anti-apoptotic protein MCL-1 levels or SRC phosphorylation (Figure 4A); both phenomena were exclusively observed in PANC-1 cells in response to FGF2 stimulation. MCL-1/SRC is a widely recognized signaling pathway regulating cell invasion in several tumors [30,31], including PDAC [32], for which we recently demonstrated a dependence on the FGFR2c/PKCε axis [11]. Therefore, we checked the possibility that TRPA1 could also contribute to the enhancement of the MCL/SRC downstream effect of cell invasion. Using the in vitro assay of Matrigel pre-coated Transwell Boyden chambers, we found that TRPA1 depletion was sufficient to attenuate the significant increase in cell invasion in response to FGF2, an event that was visible only in PANC-1 cells (Figure 4B).

To be able to discriminate between a pore-dependent or a pore-independent function of TRPA1, we took advantage of the use of A-967079, which is a highly selective antagonist of TRPA1 [33], previously used to induce an efficient inhibition of TRPA1 in the same cellular model of PANC-1 highly expressing FGFR2c [17]. In our experiments, the same experimental conditions were applied to ensure the efficient inhibition of the channel in PANC-1, as previously confirmed by calcium microfluorimetry assays [17].

**Figure 5:**
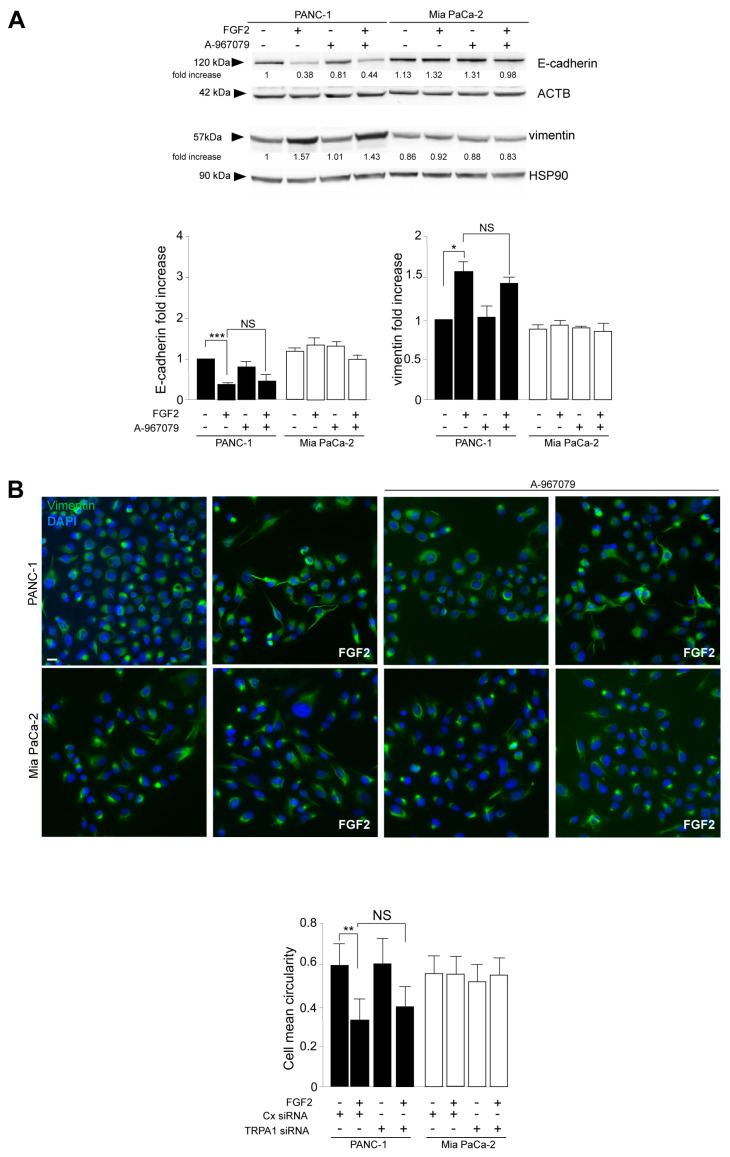
The channel-dependent function of TRPA1 is not required for its contribution to both the FGF2-mediated enhancement of the EMT signature and cell invasion. PANC-1 and MIA PaCa-2 cells were pre-treated with the selective TRPA1 pore function inhibitor (A-967079) for 1 h and then left untreated or stimulated with FGF2 for 24 h, as shown above. (**A**) Western blot analysis showed that the inhibition of the TRPA1 pore function did not impact PANC-1 cell response to FGF2 in terms of E-cadherin/vimentin modulation toward EMT enhancement. The densitometric analysis and the statistical evaluation were performed as reported in the Materials and Methods section; * *p* < 0.05; *** *p* < 0.001. (**B**) Immunofluorescence analysis showed that the effects of FGF2 on PANC-1 cells, in terms of changes in cell morphology and the increased intensity of vimentin immunostaining, were not affected by the TRPA1 inhibitor. Bar: 10 μm. Quantitative analysis of cell circularity and the statistical evaluation were performed as reported above; ** *p* < 0.01.

**Figure 6:**
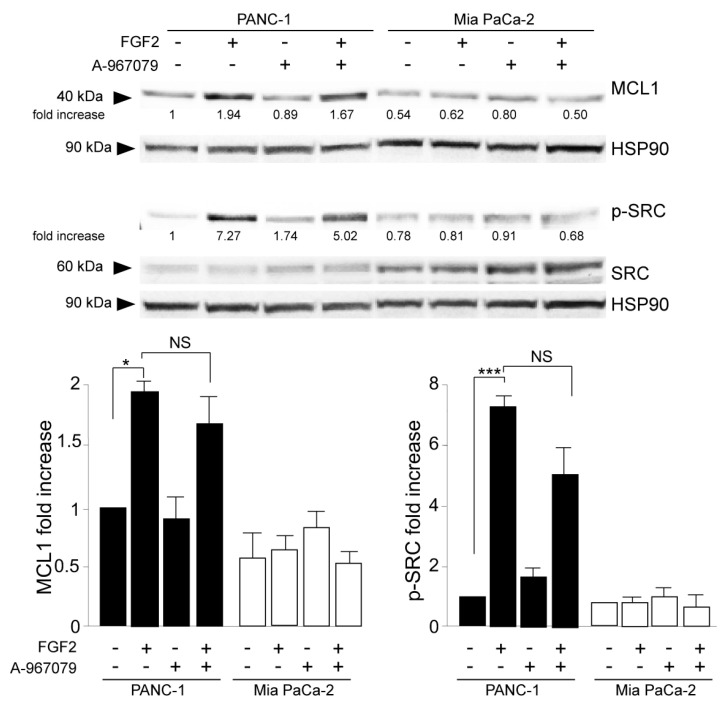
The channel-dependent activity of TRPA1 is dispensable for its involvement in cell invasion. PANC-1 and MIA PaCa-2 cells were pre-treated with the selective TRPA1 pore function inhibitor (A-967079) for 1 h and then left untreated or stimulated with FGF2 for 24 h, as shown above. Western blot analysis showed that the TRPA1 inhibitor did not impact the increase in MCL-1 levels or SRC phosphorylation, observed only in PANC-1 cells after FGF2 stimulation. The densitometric analysis and the statistical evaluation were performed as shown above; * *p* < 0.05; *** *p* < 0.001.

Parallel biochemical and immunofluorescence analyses demonstrated that the specific inhibition of pore function in TRPA1 does not impact PANC-1 cell response to FGF2 in terms of E-cadherin, vimentin (Figure 5A), α-SMA (Supplementary Figure S3A) modulation toward EMT enhancement, induction of the EMT-related transcription factor Snail 1 (Supplementary Figure S3B), changes in cell morphology, and the increase in vimentin immunostaining (Figure 5B). In a comparable way, the inhibition of TRPA1 pore function did not affect the activation of the MCL1/SRC pathway, resulting in it being ineffective towards the FGF2-induced increase in MCL1 protein expression and SRC phosphorylation (Figure 6). This evidence indicated that all the effects induced by TRPA1 depletion on the oncogenic outcomes established by the FGFR2c/PKCε axis can be attributable to a pore-independent function, the nature of which remains a challenging topic to be investigated in the future.

## 4. Discussion

Pancreatic ductal adenocarcinoma (PDAC) is a malignant carcinoma whose frequent detection at advanced stages limits the treatment to systemic chemotherapy, the results of which are poorly effective because of resistance development [1,2,3,34]. Recent advances in the knowledge of molecular profiles and cancer biology, obtained by detailed analysis of tumor samples and the use of genetically engineered mouse models (GEMMs), led to the identification of several PDAC subtypes [2,35,36]. This wide diversity of PDAC encouraged researchers to focus on subtype-specific target approaches and to find evidence to support their potential. However, despite the encouraging preclinical findings, clinical trials were still very modest because of the development of resistance, possibly attributable to the activation of multiple compensatory signaling networks whose molecular players remain unknown [2].

Like target therapy, immunotherapy is also still largely unencouraging [37,38] because of the spatial organization of the PDAC stroma, where pro-tumor cancer-associated fibroblasts (CAF), macrophages, and a dense extracellular matrix surround the tumor cells, crosstalking with them and making the tumor core inaccessible to T cells [39,40]. Therefore, to “unravel” the tumor/stromal supportive network, identifying the molecular players involved in it will help identify the potential vulnerabilities of PDAC. 

Since the landscape of the tumor/stroma crosstalk FGF/FGFR appeared to play a central role [6,7,8], we very recently demonstrated the specific involvements of the mesenchymal variant of FGFR2 (FGFR2c) and its downstream PKCε aberrant axis in the enhancement of the EMT-profile and the tumorigenic features of PDAC-derived cells [10,11]. Therefore, in this work, we further advanced the knowledge of FGFR2c-mediated tumorigenesis, investigating the possible contribution of Ca^2+^ preferential cationic channel TRPs. In fact, even if the specific role of TRPs in cancer remains to be better clarified, accumulating observations have suggested that in establishing a cancer hallmark, receptor tyrosine kinases, including FGFRs, can enter in a crosstalk with them [12], and the channel can contribute via either a channel-dependent or channel-independent function [13,14]. Since TRPA1, TRPC1, and TRPM8 are the TRPs that are the most overexpressed in PDAC [17,24,25,26], our investigation focused on them. Biochemical data demonstrated that among the three analyzed TRPs, only TRPA1 was downregulated at protein levels by either the shut-off of the FGFR2/PKCε axis via stable gene silencing or its sustained activation by prolonged FGF2 stimulation. The repressive effects were observed exclusively in PANC-1 cells highly expressing FGFR2c, further strengthening the idea of their dependence on high FGFR2c expression and the consequent aberrant signaling. Then, the immunofluorescence analysis of the intracellular relocalization of both FGFR2c and the TRPA1 and the biochemical check of their protein amounts in response to the alternative block of the proteasomal or the lysosomal degradative pathways revealed that FGFR2c and TRPA1 follow different fates in response to FGF2 stimulation. However, the expression of FGFR2c and its moderate signaling are required for TRPA1 stability. These findings further strengthen the possibility of their functional dependence. 

**Figure 7:**
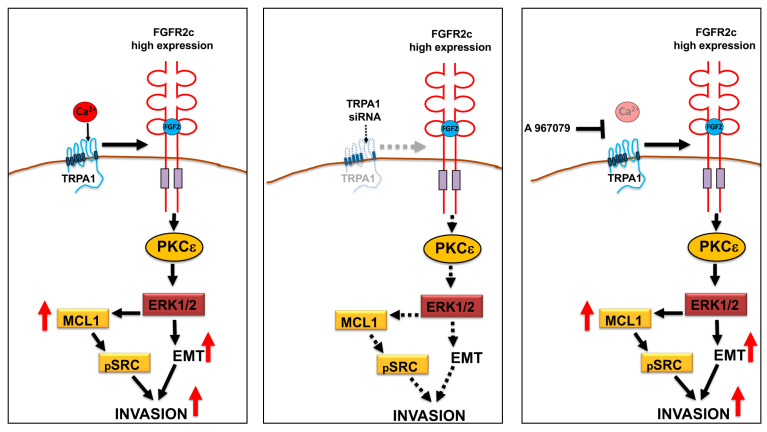
Schematic drawing of the impact of TRPA1 depletion or pore-function inhibition on FGR2c/PKCε signaling and tumorigenic outcomes. The depletion of TRPA1 expression by siRNA, but not its functional block using the antagonist A967079, significantly repressed the FGR2c/PKCε axis and the enhancement of EMT, as well as the MCL-1-dependent increase in cell invasion.

In the second step, the biochemical analysis performed in PDAC cell lines transiently transfected with TRPA1 siRNA to obtain TRPA1 depletion evidenced the contribution of TRPA1 expression in the activation of the oncogenic FRS2–PKCε–ERK and MTOR/S6K signaling pathways downstream from FGFR2c. In addition, focusing our attention on the FGF2-mediated modulation of the epithelial marker E-cadherin, the mesenchymal markers vimentin and α-SMA, and the induction of the EMT-related transcription factor Snail 1, as well as on changes in cell morphology compatible with an enhancement of EMT, we observed that these events, triggered exclusively in cells highly expressing FGFR2c in response to FGF2, were significantly impaired by TRPA1 depletion. A sensible repressive effect was also detectable upon the induction of the anti-apoptotic protein MCL-1 and upon the consequent phosphorylation of SRC, a signaling event that was recently proposed to contribute to the increase in cell invasiveness in response to FGF2 [11]. Indeed, cell invasion was another oncogenic hallmark whose enhancement by FGFR2c aberrant signaling was compromised by TRPA1 depletion (Figure 7).

Finally, the use of A-967079, which is a highly selective antagonist of TRPA1 [33], unequivocally demonstrated that all the effects observed in consequence of TRPA1 silencing, in terms of EMT enhancement, changes in cell morphology, and improvements in MCL1/SRC-signaling, did not require its pore function (Figure 7). The molecular mechanisms underlying this function are still unknown and are worth being investigated in the future. 

Our results are consistent with previous findings obtained in lung adenocarcinoma and suggest that cell invasion in this context involves a crosstalk between FGFR2 and TRPA1, which implies a pore-independent function of the channel [15]. In contrast, a more recent work proposed a pore-dependent function for TRPA1 in lung and breast cancer pro-survival signaling, which seems to exclude FGFR2 involvement [41]. The channel-independent role of TRPA1 in cell invasion was also recently proposed in PDAC, even if a correlation with FGFR2 expression was investigated by the authors [17].

## 5. Conclusions

In the landscape of the still-debated role of TRPs in cancer, our data support the line of thought proposing the important contribution of TRPs in RTK signaling dysregulation during carcinogenesis. Our data encourage the consideration of TRPA1, the mesenchymal FGFR2c variant, and its hub signaling molecule PKCε as new molecular targets for precision oncology approaches to this recalcitrant cancer, which we hope will benefit an ever-larger group of patients.

## Data Availability

All the data are provided in the article.

## Supplementary Materials

The following supporting information can be downloaded at: https://www.mdpi.com/article/10.3390/cancers16030609/s1, Figure S1. Efficiency of FGFR2 or PKCe stable depletion by specific shRNAs. PANC-1 and Mia PaCa-2 cells were stably transfected with Bek/FGFR2 short hairpin RNA (shRNA), with PKCε shRNA, to ob-tain stable protein depletion. Unrelated shRNA (Cx shRNA) was used as negative control. (A) Mo-lecular analysis by real time RT-PCR shows that untransfected PDAC cell lines express divergent levels of the mesenchymal FGFR2c isoform, and negligible levels of the epithelial FGFR2b variant. Results are reported as mean ± SD from three different experiments in triplicate. ** *p* < 0.01 (B) The efficiency of the gene silencing by shRNAs was assessed by Western blot analysis. For the densi-tometric analysis, the values from 3 independent experiments were normalized, expressed as fold increase and reported in graph as mean values ± standard deviation (SD). Student’s t test was per-formed, and significance levels have been defined as *p* < 0.05: ** *p* < 0.01, *** *p* < 0.001. Figure S2. TRPA1 depletion impacts on the increase of the expression of a-SMA marker and on the induction of the EMT-related transcription factor Snail1 in response to FGF2. PANC-1 and Mia PaCa-2 cells were transfected with TRPA1 siRNA or control (Cx) siRNA and left untreated or stimulated with FGF2 for 24 h. (A) Western blot analysis shows the conteracting effects of TRPA1 depletion on the increase of a-SMA only in PANC-1 cells. The densitometric analysis, and the statistical evaluation were performed as reported in materials and methods: *** *p* <0.001 (B) Molecular analysis by real time RT-PCR shows that, only in PANC-1 cells, the silencing of TRPA1 also counteracts the induc-tion of Snail1. Results are reported as mean ± SD from three different experiments in triplicate. Statistical analysis was performed as reported in materials and methods: *** *p* < 0.001. Figure S3. The inhibition of the pore function of TRPA1 does not impact on the increase of a-SMA and Snail1 induced by FGF2. PANC-1 and Mia PaCa-2 cells were pre-treated with A-967079 inhibitor and then left untreated or stimulated with FGF2 for 24 h. (A) Western blot analysis shows that the inhibitor has no influence on the increase of a-SMA, visible in PANC-1 samples in response to FGF2. The densitometric analysis and the statistical evaluation were performed as reported in ma-terials and methods: *** *p* < 0.001. (B) Real time RT-PCR shows that A-967079 does not affect the in-ductive effect of FGF2 on Snail1 expression. Results are reported as mean ± SD from three differ-ent experiments in triplicate. Statistical analysis was performed as reported in materials and methods: ** *p* < 0.01, *** *p* <0.001.
