# Supplementary material for: TRPA1 Contributes to FGFR2c Signaling and to Its Oncogenic Outcomes in Pancreatic Ductal Adenocarcinoma-Derived Cell Lines
Source: Cancers (Basel). 2024 Jan 31;16(3):609. doi: 10.3390/cancers16030609 (PMC10854535; doi:10.3390/cancers16030609)
Supplement: Supplementary file 1 [file cancers-16-00609-s001.zip › cancers-2785804-supplementary.pdf]

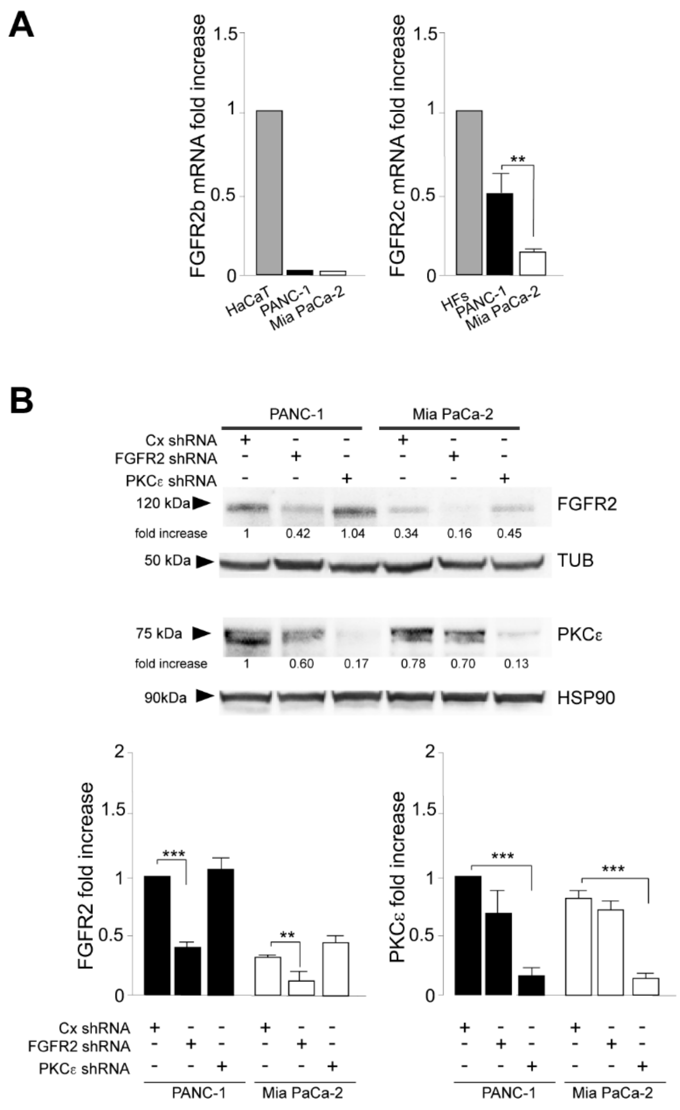

Supplementary Figure S1.

Efficiency of FGFR2 or PKCε stable depletion by specific shRNAs.

PANC-1 and Mia PaCa-2 cells were stably transfected with Bek/FGFR2 short hairpin RNA (shRNA), with PKCε shRNA, to obtain stable protein depletion. Unrelated shRNA (Cx shRNA) was used as negative control. (A) Molecular analysis by real time RT-PCR shows that untransfected PDAC cell lines express divergent levels of the mesenchymal FGFR2c isoform, and negligible levels of the epithelial FGFR2b variant. Results are reported as mean ± SD from three different experiments in triplicate. ** $p < 0.01$ (B) The efficiency of the gene silencing by shRNAs was assessed by Western blot analysis. For the densitometric analysis, the values from 3 independent experiments were normalized, expressed as fold increase and reported in graph as mean values ± standard deviation (SD). Student's t test was performed, and significance levels have been defined as $p < 0.05$: ** $p < 0.01$, *** $p < 0.001$.

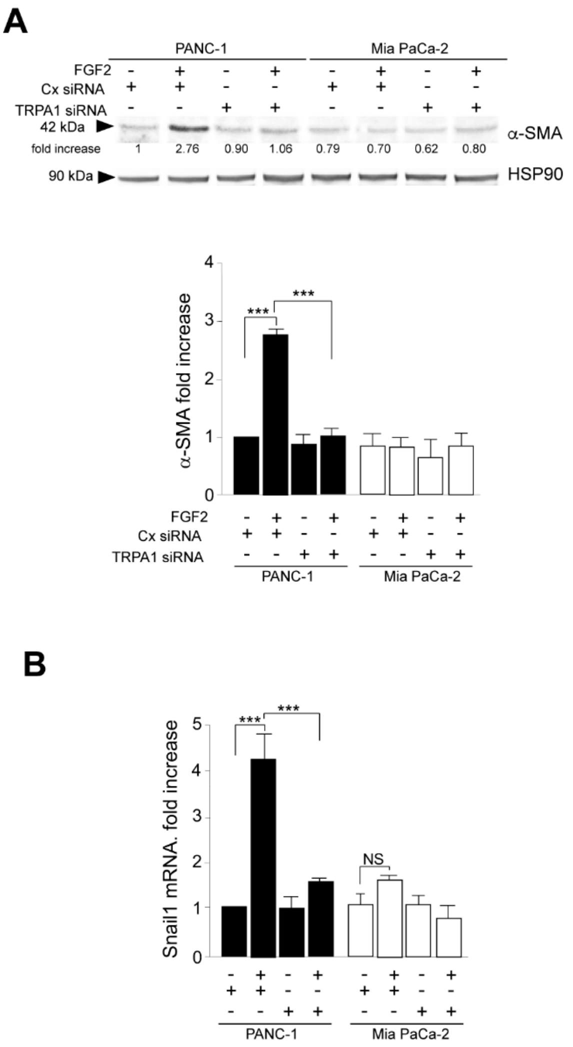

Supplementary Figure S2.

TRPA1 depletion impacts on the increase of the expression of α-SMA marker and on the induction of the EMT-related transcription factor Snail1 in response to FGF2.

PANC-1 and Mia PaCa-2 cells were transfected with TRPA1 siRNA or control (Cx) siRNA and left untreated or stimulated with FGF2 for 24h. (A) Western blot analysis shows the conteracting effects of TRPA1 depletion on the increase of α-SMA only in PANC-1 cells. The densitometric analysis, and the statistical evaluation were performed as reported in materials and methods: ***p <0.001 (B) Molecular analysis by real time RT-PCR shows that, only in PANC-1 cells, the silencing of TRPA1 also counteracts the induction of Snail1. Results are reported as mean ± SD from three different experiments in triplicate. Statistical analysis was performed as reported in materials and methods: ***p <0.001.

A

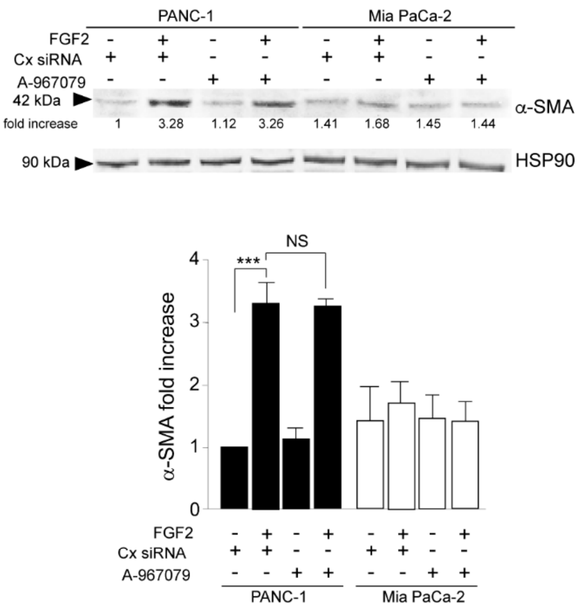

B

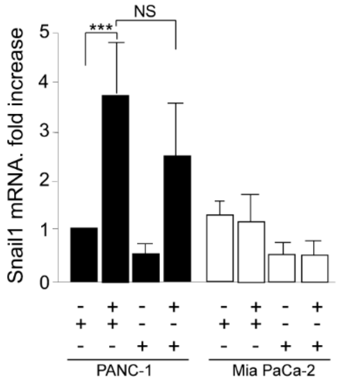

Supplementary Figure S3.
The inhibition of the pore function of TRPA1 does not impact on the increase of α-SMA and Snail1 induced by FGF2.
PANC-1 and Mia PaCa-2 cells were pre-treated with A-967079 inhibitor and then left untreated or stimulated with FGF2 for 24h. (A) Western blot analysis shows that the inhibitor has no influence on the increase of α-SMA, visible in PANC-1 samples in response to FGF2. The densitometric analysis and the statistical evaluation were performed as reported in materials and methods: ***p <0.001(B) Real time RT-PCR shows that A-967079 does not affect the inductive effect of FGF2 on Snail1 expression. Results are reported as mean ± SD from three different experiments in triplicate. Statistical analysis was performed as reported in materials and methods:** ***p <0.001.

1A

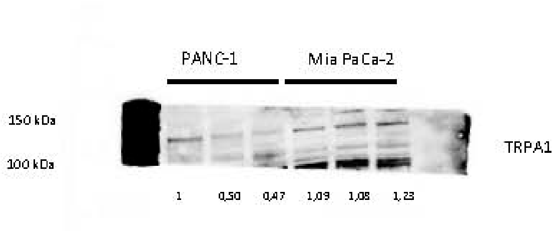
PANC-1
Mia PaCa-2
150 kDa
100 kDa
TRPA1
1
0,50
0,47
1,09
1,08
1,23

1A

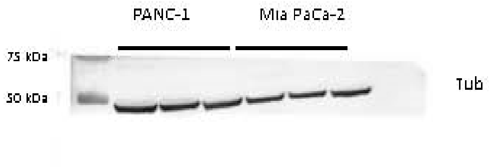
PANC-1
Mia PaCa-2
75 kDa
50 kDa
Tub

1A

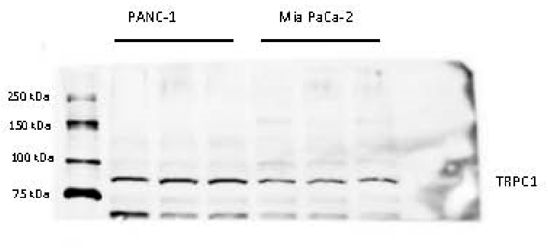
PANC-1
Mia PaCa-2
250 kDa
150 kDa
100 kDa
75 kDa
TRPC1

1A

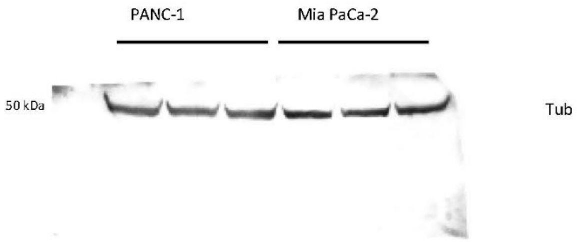
PANC-1
Mia PaCa-2
50 kDa
Tub

1A

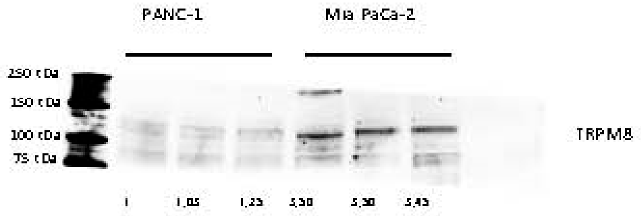
PANC-1
Mia PaCa-2
250 kDa
150 kDa
100 kDa
75 kDa
TRPM8
1
1,05
1,25
3,50
3,30
3,45

1A

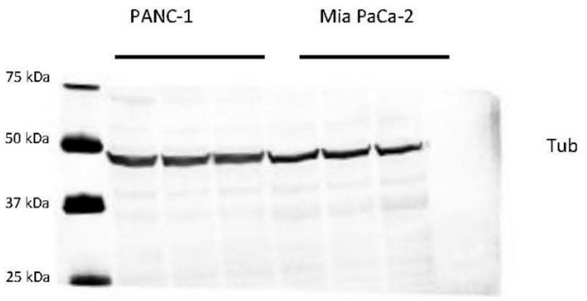
PANC-1
Mia PaCa-2
75 kDa
50 kDa
37 kDa
25 kDa
Tub

1C

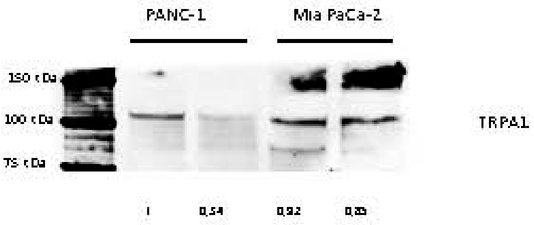
PANC-1
Mia PaCa-2
150 kDa
100 kDa
75 kDa
TRPA1
1
0,54
0,92

1C

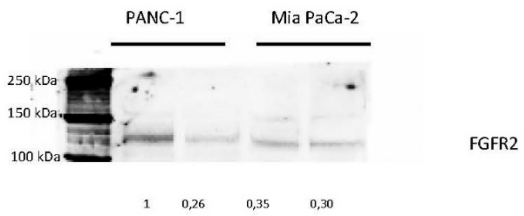
PANC-1
Mia PaCa-2
250 kDa
150 kDa
100 kDa
FGFR2
1
0,26
0,35
0,30

1C

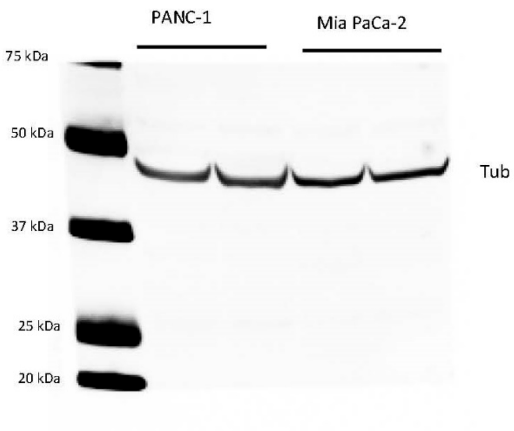
PANC-1
Mia PaCa-2
75 kDa
50 kDa
37 kDa
25 kDa
20 kDa
Tub

1D

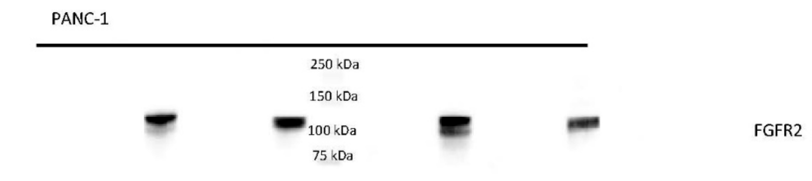
PANC-1
250 kDa
150 kDa
100 kDa
75 kDa
FGFR2

1D

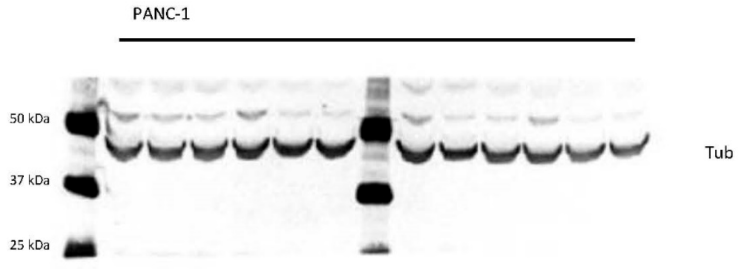
PANC-1
50 kDa
37 kDa
25 kDa
Tub

1D

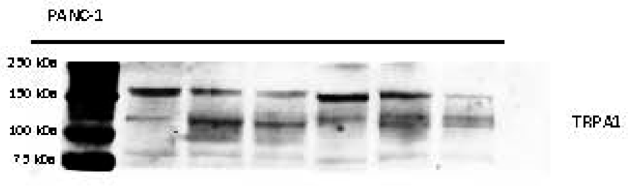
PANC-1
250 kDa
150 kDa
100 kDa
75 kDa
TRPA1

1D

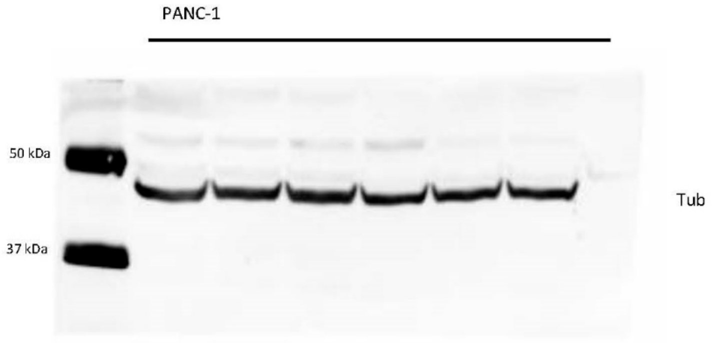
PANC-1
50 kDa
37 kDa
Tub

2E

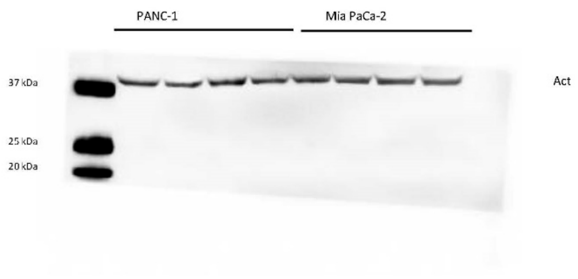
PANC-1
Mia PaCa-2
37 kDa
25 kDa
20 kDa
Act

2E

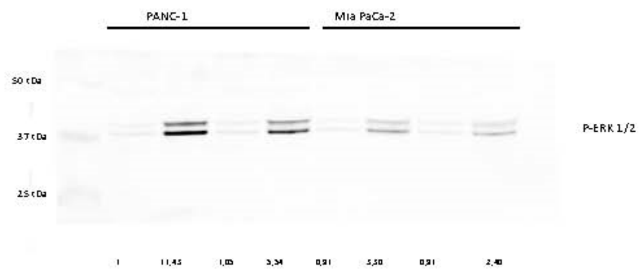
PANC-1
Mia PaCa-2
50 kDa
37 kDa
25 kDa
P-ERK 1/2
1
11,45
1,05
3,34
0,91
3,50
0,91
2,40

2E

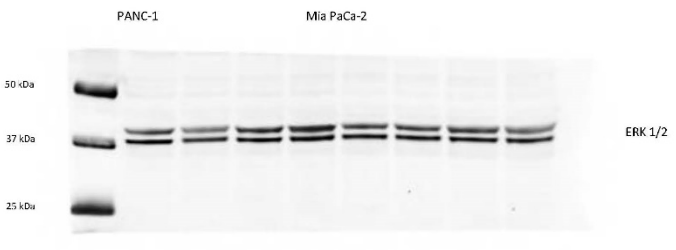
PANC-1
Mia PaCa-2
50 kDa
37 kDa
25 kDa
ERK 1/2

2E

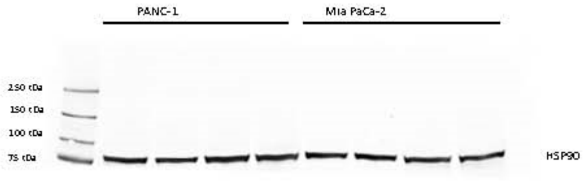
PANC-1
Mia PaCa-2
250 kDa
150 kDa
100 kDa
75 kDa
HSP90

2E

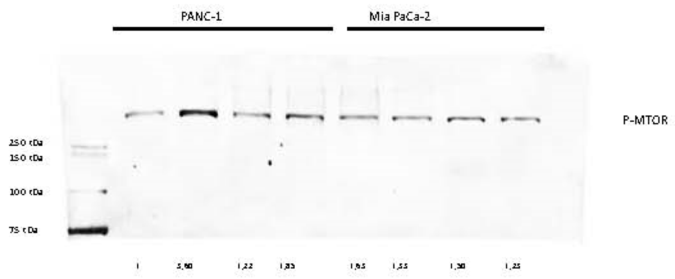
PANC-1
Mia PaCa-2
P-MTOR
250 kDa
150 kDa
100 kDa
75 kDa
1
5,60
1,22
1,85
1,65
1,55
1,30
1,25

2E

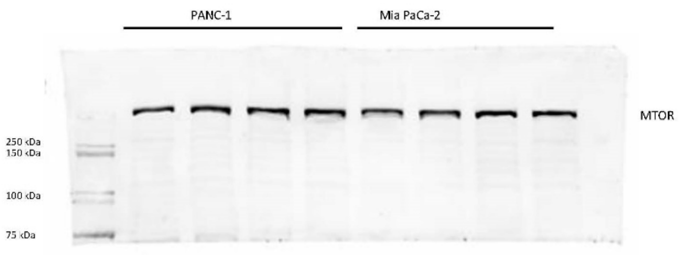
PANC-1
Mia PaCa-2
MTOR
250 kDa
150 kDa
100 kDa
75 kDa

2E

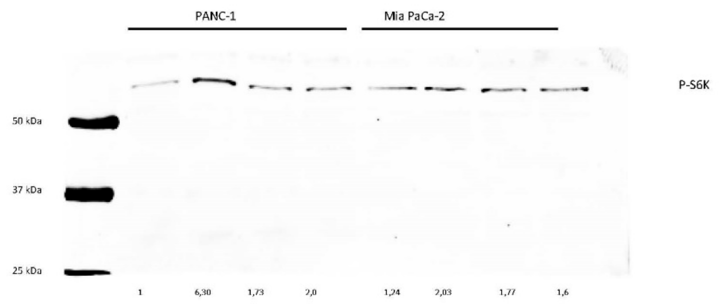
PANC-1
Mia PaCa-2
P-S6K
50 kDa
37 kDa
25 kDa
1
6,30
1,73
2,0
1,24
2,03
1,77
1,6

2E

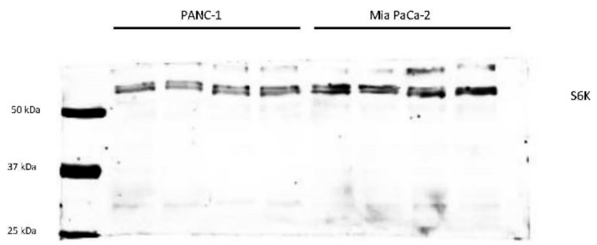
PANC-1
Mia PaCa-2
S6K
50 kDa
37 kDa
25 kDa

2E

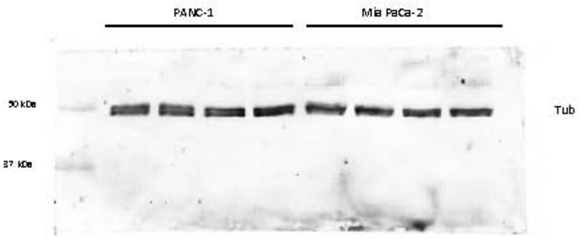
PANC-1
Mia PaCa-2
50 kDa
37 kDa
Tub

2B

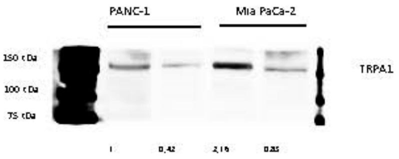
PANC-1
Mia PaCa-2
150 kDa
100 kDa
75 kDa
TRPA1
1
0,42
2,16
0,85

2B

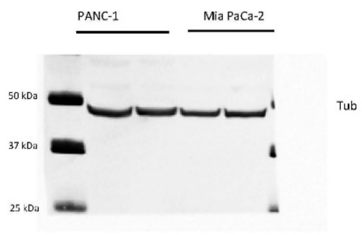
PANC-1
Mia PaCa-2
50 kDa
37 kDa
25 kDa
Tub

2D

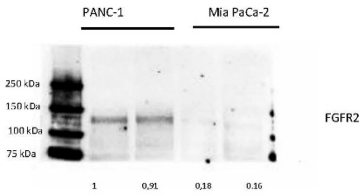
PANC-1
Mia PaCa-2
250 kDa
150 kDa
100 kDa
75 kDa
FGFR2
1
0,91
0,18
0.16

2D

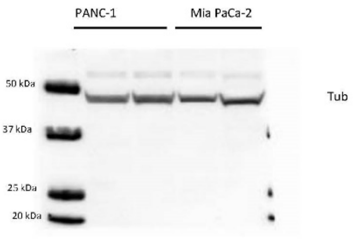
PANC-1
Mia PaCa-2
50 kDa
37 kDa
25 kDa
20 kDa
Tub

2E

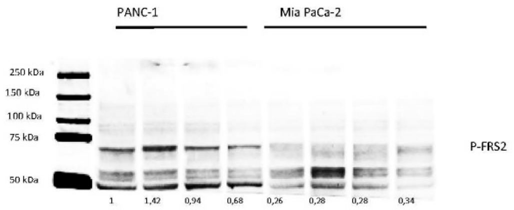
PANC-1
Mia PaCa-2
250 kDa
150 kDa
100 kDa
75 kDa
50 kDa
P-FRS2
1
1,42
0,94
0,68
0,26
0,28
0,28
0,34

2E

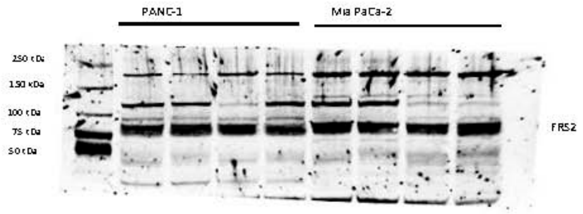
PANC-1
Mia PaCa-2
250 kDa
150 kDa
100 kDa
75 kDa
50 kDa
FRS2

2E

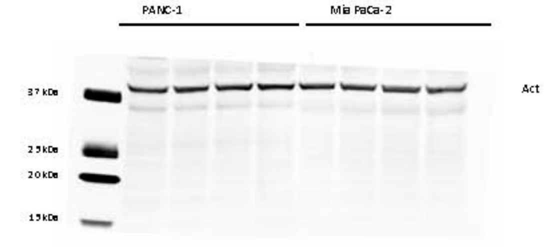
PANC-1
Mia PaCa-2
37 kDa
25 kDa
20 kDa
15 kDa
Act

3A

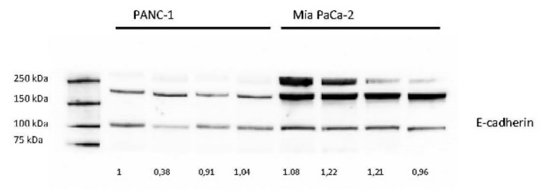
PANC-1
Mia PaCa-2
250 kDa
150 kDa
100 kDa
75 kDa
E-cadherin
1
0,38
0,91
1,04
1.08
1,22
1,21
0,96

3A

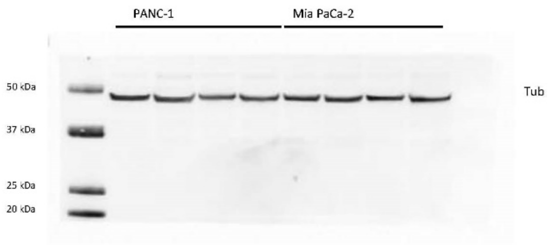
PANC-1
Mia PaCa-2
50 kDa
37 kDa
25 kDa
20 kDa
Tub

3A

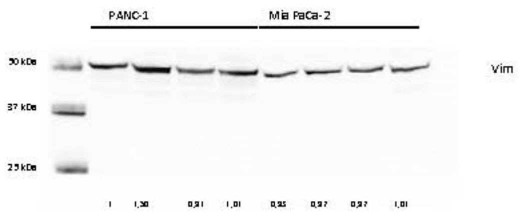
PANC-1
Mia PaCa-2
50 kDa
37 kDa
25 kDa
Vim
1
1,30
0,91
1,01
0,95
0,97
0,97
1,01

3A

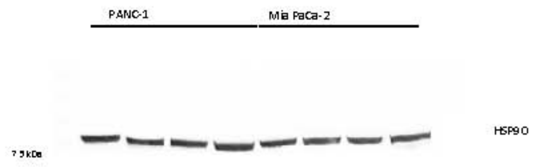
PANC-1
Mia PaCa-2
HSP90
75 kDa

2E

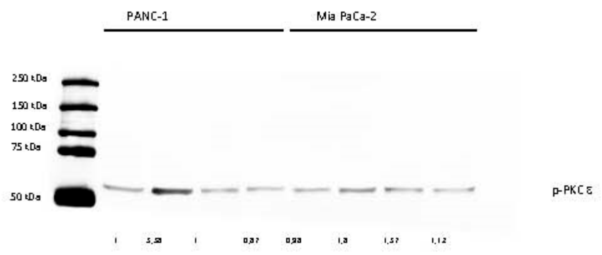
PANC-1
Mia PaCa-2
250 kDa
150 kDa
100 kDa
75 kDa
50 kDa
p-PKC ε
1
1
0,87
1,57
1,12

2E

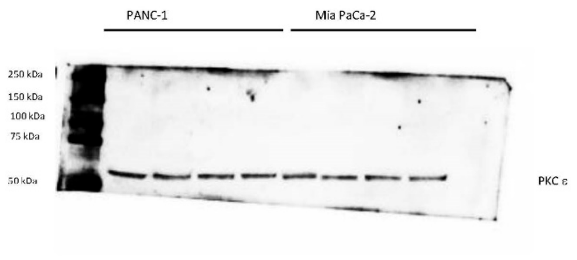
PANC-1
Mia PaCa-2
250 kDa
150 kDa
100 kDa
75 kDa
50 kDa
PKC ε

4A

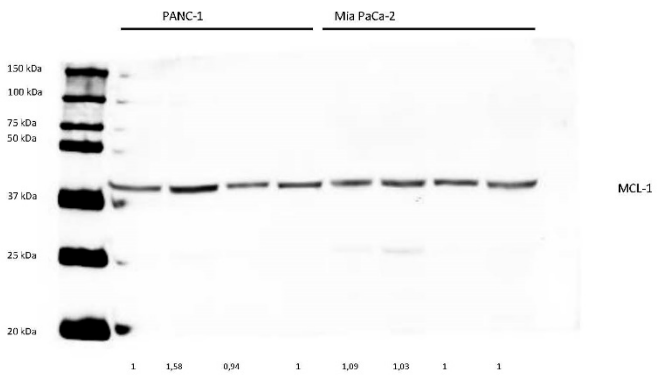
PANC-1
Mia PaCa-2
150 kDa
100 kDa
75 kDa
50 kDa
37 kDa
25 kDa
20 kDa
MCL-1
1
1,58
0,94
1
1,09
1,03
1
1

4A

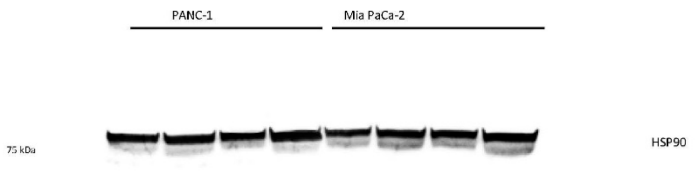
PANC-1
Mia PaCa-2
75 kDa
HSP90

4A

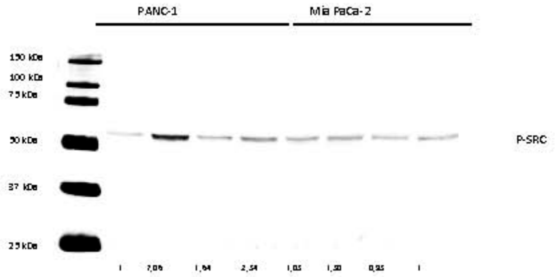
PANC-1
Mia PaCa-2
150 kDa
100 kDa
75 kDa
50 kDa
37 kDa
25 kDa
P-SRC
1
7,06
1,64
2,54
1,05
1,50
0,95
1

5A

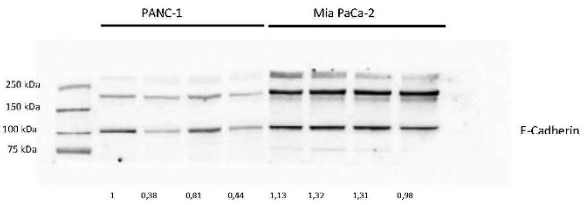
PANC-1
Mia PaCa-2
250 kDa
150 kDa
100 kDa
75 kDa
E-Cadherin
1
0,38
0,81
0,44
1,13
1,32
1,31
0,98

5A

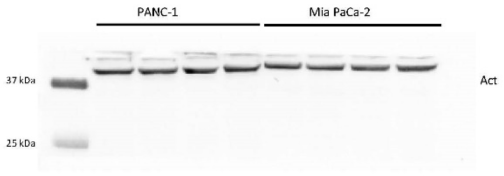
PANC-1
Mia PaCa-2
37 kDa
25 kDa
Act

5A

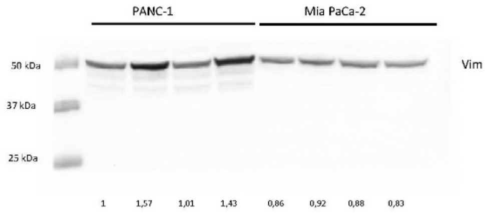
PANC-1
Mia PaCa-2
50 kDa
37 kDa
25 kDa
Vim
1
1,57
1,01
1,43
0,86
0,92
0,88
0,83

5A

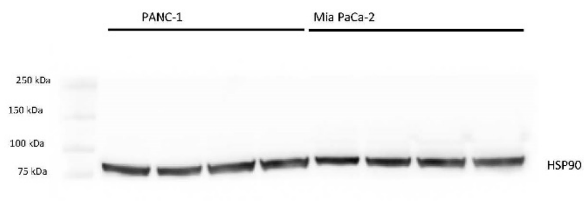

PANC-1
Mia PaCa-2
250 kDa
150 kDa
100 kDa
75 kDa
HSP90

6A

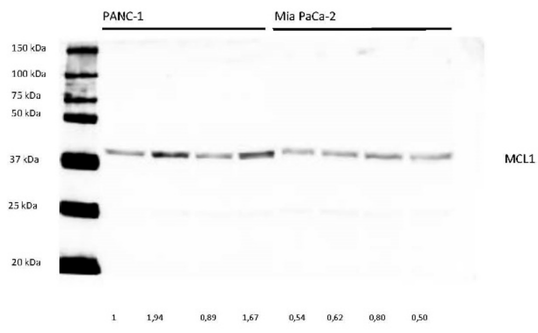

PANC-1
Mia PaCa-2
150 kDa
100 kDa
75 kDa
50 kDa
37 kDa
25 kDa
20 kDa
MCL1
1
1,94
0,89
1,67
0,54
0,62
0,80
0,50

6A

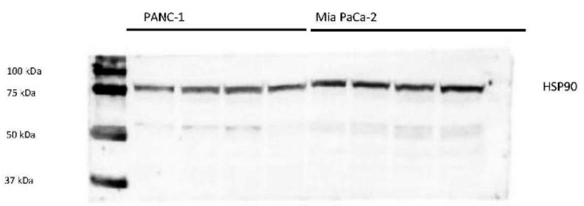
PANC-1
Mia PaCa-2
100 kDa
75 kDa
50 kDa
37 kDa
HSP90

6A

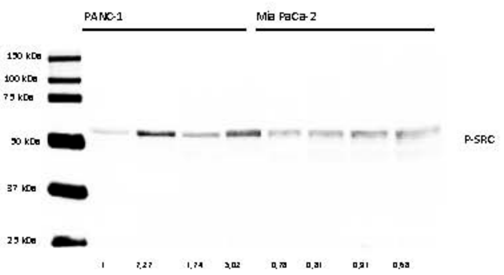
PANC-1
Mia PaCa-2
150 kDa
100 kDa
75 kDa
50 kDa
37 kDa
25 kDa
P-SRC

6A

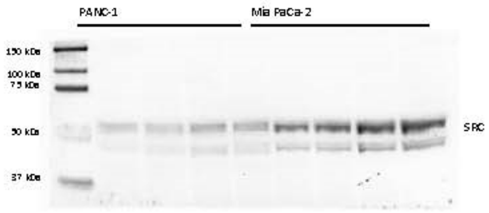
PANC-1
Mia PaCa-2
150 kDa
100 kDa
75 kDa
50 kDa
37 kDa
SRC

Suppl B

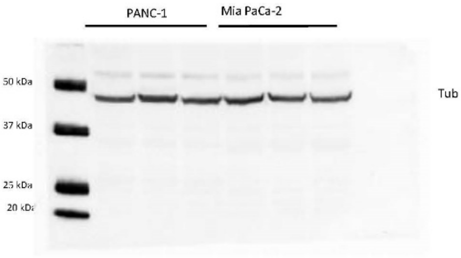
PANC-1
Mia PaCa-2
50 kDa
37 kDa
25 kDa
Tub

Suppl B

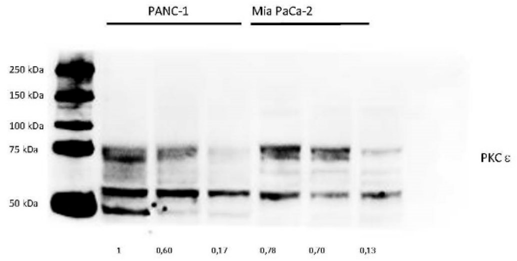

Suppl B

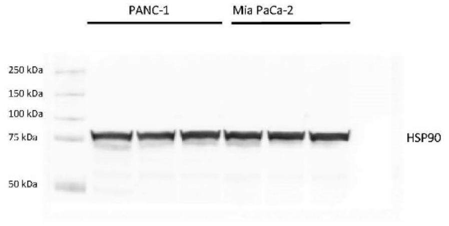

Suppl B

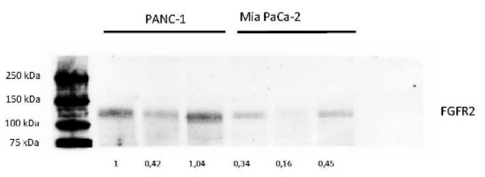

Suppl.2

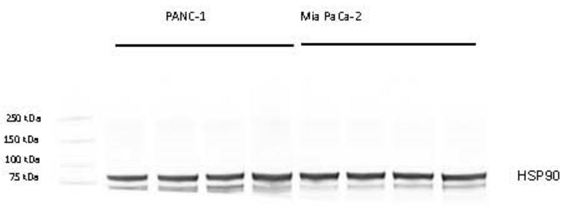

Suppl.2

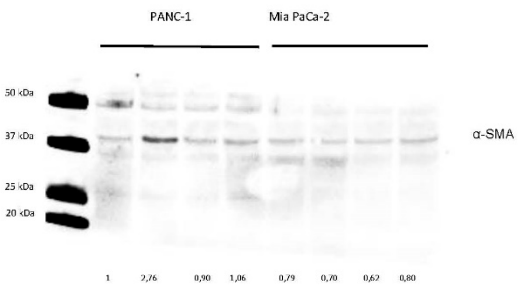
PANC-1
Mia PaCa-2
50 kDa
37 kDa
25 kDa
20 kDa
α-SMA
1
2,76
0,90
1,06
0,79
0,70
0,62
0,80

4A

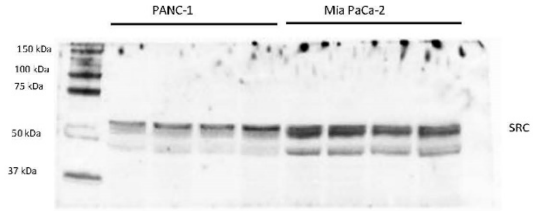
PANC-1
Mia PaCa-2
150 kDa
100 kDa
75 kDa
50 kDa
37 kDa
SRC

1A

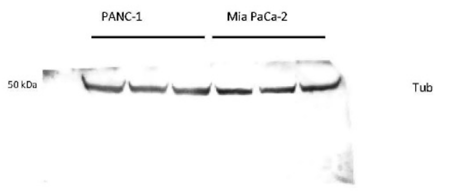
PANC-1
Mia PaCa-2
50 kDa
Tub

Suppl.3

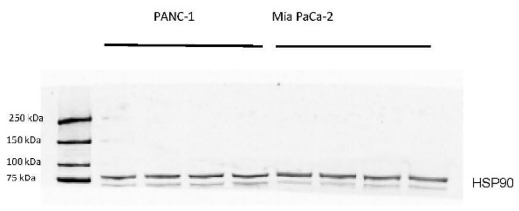
PANC-1
Mia PaCa-2
250 kDa
150 kDa
100 kDa
75 kDa
HSP90

Suppl.3

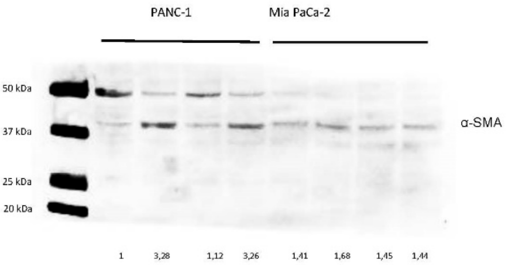

PANC-1
Mia PaCa-2
50 kDa
37 kDa
25 kDa
20 kDa
α-SMA
1
3,28
1,12
3,26
1,41
1,68
1,45
1,44

1A

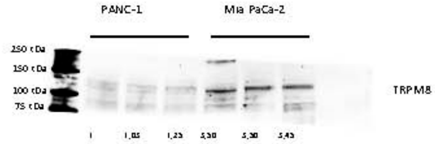

PANC-1
Mia PaCa-2
250 kDa
150 kDa
100 kDa
75 kDa
TRPM8
1
1,05
1,25
5,50
5,30
5,45

1A

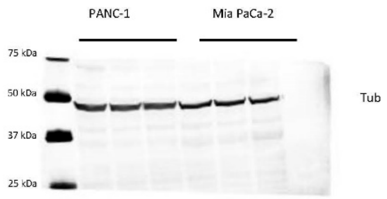
PANC-1
Mia PaCa-2
75 kDa
50 kDa
37 kDa
25 kDa
Tub

1C

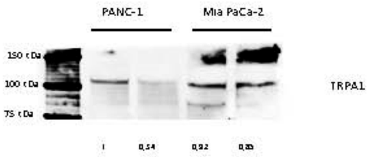
PANC-1
Mia PaCa-2
150 kDa
100 kDa
75 kDa
TRPA1
1
0,54
0,92
